# Response to Comment on Giuseppe Genchi et al. Mercury Exposure and Heart Diseases. *Int. J. Environ. Res. Public Health* 2017, 14, 74

**DOI:** 10.3390/ijerph14070761

**Published:** 2017-07-12

**Authors:** Giuseppe Genchi, Maria Stefania Sinicropi, Alessia Carocci, Graziantonio Lauria, Alessia Catalano

**Affiliations:** 1 Dipartimento di Farmacia e Scienze della Salute e della Nutrizione, Università della Calabria, 87036 Arcavacata di Rende (Cosenza), Italy; giuseppe.genchi@unical.it (G.G.); glauria@unical.it (G.L.); 2 Dipartimento di Farmacia-Scienze del Farmaco, Università degli Studi di Bari “A. Moro”, 70125 Bari, Italy; alessia.catalano@uniba.it

We are grateful to Paknahad and coauthors for giving us the opportunity to consider the interesting papers by Kursun et al., Mortazavi S.M.J. et al., Mortazavi G. et al., Shahidi et al., Yilmaz et al., about the effects of electromagnetic fields (EMFs) produced by magnetic resonance imaging (MRI) or those generated by other sources such as mobile phones, mobile base stations, cordless phones, Wi-Fi routers, radio and TV broadcasting, and X-rays which can increase the mercury release from amalgam fillings [1,2,3,4,5,6]. The effects of these EMFs allow the release of mercury, which can adversely affect patients’ health, aside from being a high environmental pollutant. Nevertheless, “the mercury levels which normally can be released from amalgam fillings, even in the presence of EMFs, are not high enough to cause toxicity”, as Paknahad and coauthors assert in their Letter to Editor [7]. Nevertheless, we agree with the authors in that pregnant women, children, and hypersensitive members of the population may be subject to the toxicity of mercury.

Moreover, it should be underlined that nowadays, the harmful effects of mercury released from amalgam fillings are generally accepted throughout the world, as it has been recognized several decades ago by the OMS, which recommends avoiding the use of mercury in amalgam fillings. This recommendation is followed in many Western countries including European countries where less dangerous and more aesthetic filling systems are used. Thus, the number of individuals whose caries are treated with mercury amalgam is getting lower.

We apologize to the authors of the letter for not having mentioned their works in our review. Anyway, when writing a review it may happen that papers, although very interesting, may not be taken into account, as they are not considered relevant to the context of the paper.

We would thank the authors for their positive criticism and feedback, and the editor for giving us the opportunity to provide a reply to the letter.
